# Bouncing bones—ancient wisdom meets modern science in a new take on locomotion

**DOI:** 10.3389/fphys.2024.1432410

**Published:** 2024-09-30

**Authors:** Stephen M. Levin, Susan Lowell de Solórzano

**Affiliations:** Ezekiel Biomechanics Group, McLean, VA, United States

**Keywords:** biotensegrity, processual biology, ecological psychology, walking and running, muscle and bone physiology, T’ai Chi, human movement, tensegrity

## Abstract

Recognizing that conventional understanding of animal and human locomotion is based on a dated and reductionist machine modeling of organisms, we set out to create a theory of locomotion by reasoning from first principles. We center on the constraints necessitated by 1) the 2nd law of thermodynamics, 2) the theory of evolution, 3) a systems science view of organisms, and 4) the laws of motion, but we also look for compatibility these constraints might find in emerging areas of scientific inquiry (ecological psychology, processual biology, soft matter, biotensegrity), and in the wisdom embedded in various movement traditions and ancient philosophy. Applying and synthesizing these, we propose an updated “bouncing bones” (BB) model for walking and running, which corresponds with maximum efficiency and conservation of energy.

## Highlights

• Abandoning machine models for locomotion that have persisted for centuries, we reason from first principles to derive a model more compatible with the current science.
• We underscore that, following the second law of thermodynamics and evolutionary theory, organisms maximize for energy efficiency.
• Recognizing a systems science perspective, we see organisms as non-fractionable synergistic systems operating in states far-from-equilibrium.
• Following Newton’s laws, we recognize that during locomotion, the body’s center of mass (CoM) glides over the (briefly) stationary and essentially shear-free foot, continuing its unimpeded movement.
• We propose that in locomotion, the bones are the body’s main springs.
• Following physics, movement of a body functions as if all its mass were centered in its center of mass (CoM).
• Load transfer occurs when the CoM is directly over the the center of base and the GRF is directed straight out from the center of the earth.
• Muscles, operating as turnbuckles, assist in transferring GRF to the bones, thereby loading our springy bones.
• We put the above points together to propose a, “bouncing bones” (BB) hypothesis for human and animal locomotion, and find support for our BB theory and its foundations in the practical wisdom of movement traditions and ancient philosophy.

## 1 Introduction

Betting against the second law of thermodynamics is a sucker’s bet — there is no way of winning.

But, it is a sure bet that Mother Nature made certain from the start that biological organisms, their structure and mechanics, conform to this rule: an organism dealing with its external world will always seek to operate at maximum efficiency, just as all self-assembling systems do. Nonetheless, conventional biomechanics sidesteps this physical and biological imperative, as well as a few other accepted principles. It is time these were addressed.

In addition to guiding biological function, the second law of thermodynamics (*2LT*) also dictates evolution, ensuring that the least energy-consuming propositions will always win out (Schreiber and Gimbel, 2010; Gracovetsky, 2018). This suggests that, if we can conceive of a more energy efficient way for an organism to interface with its external world, Mother Nature is going to be way ahead of us. After all, she has been working on it for nearly 4 billion years.

Systems science tells us that organisms organize themselves far from equilibrium, and (obeying the second law of thermodynamics/2LT again) they will always self-assemble in the most energy conserving manner (Bertalanffy, 1969; Fultot et al., 2019; Turvey, 2019). Although 2LT leads to *disorder* in the inanimate world, for living organisms operating as far-from-equilibrium systems, entropy can actually be an organizing principle, and may be the property that makes needed complexity possible (Bertalanffy, 1969; Geng et al., 2019; Prigogine and Nicolis, 1985; Strogatz, 2021). When it comes to living organisms, the second law of thermodynamics, the theory of evolution, and systems science all go hand in hand. They are also at odds with the long-held practice of modeling organisms as machines.

In 1630, René Descartes wrote, “I suppose the body to be just a statue or a machine made of earth,” (Descartes and Gaukroger, 1998). His idea stuck, and in the next century the term “organism” emerged, with, as Lewontin (2000) points out, machine implications in its very etymology. Despite the fact that organisms are not machines (Nicholson, 2013; Turvey, 2019; Dupré, 2022), the view of the body as an engineered machine was reinforced by Borelli’s de Motu Animalium (1680) and de la Mettrie’s *L’homme Machine* (Mettrie, 1747), and has been a dominant theme for centuries.

Nicholson, who, along with Dupré and others advocate a processual perspective for biology, calls the machine conception of the organism (MCO), “one of the most pervasive metaphors in modern biology,” and points out that organismic models based on machines are incompatible with the second law of thermodynamics: “it is when we consider how organisms conform to it (the second law of thermodynamics) that the MCO absolutely breaks down” (Nicholson, 2018).

Ancient and practical movement wisdom, on the other hand, tends to align with holistic, systems views of organisms and 2LT. For example, T’ai Chi Ch’uan is recognized as a repository of health and movement wisdom, with over 500 studies to its credit, and has been called, “medication in motion,” by Harvard Health (Harvard Health, 2010; Huston and McFarlane, 2016). The T’ai Chi Ch’uan classic texts dictate that, rather than exerting muscular force, the goal is “least effort” or *Wei wu wei (“doing not doing”)*, relating to states of effortless flow often reported by top athletes, or states of ease and effortless cultivated in dance (Todd, 1977; Jackson and Csikszentmihalyi, 1999; Levin, 2022; Itcca, 2023). Those who practice internal martial arts (such as T’ai Chi), gymnastics, dance, acrobatics, etc. instinctively know this to be the case. Should it surprise us that traditional wisdom and the experience of movement experts is in keeping with 2LT and a systems view of organisms?

Some will question whether the inclusion of ancient, practical and traditional wisdom is a valid scientific approach. Particularly when indigenous-led or traditional wisdom repositories dovetail with more modern scientific outlooks, it would seem prudent to seriously consider them regarding their value. Venerable teachings represent countless hours of active practical research by innumerable people, informing the collective wisdom in a given field. That data is valid (if not traditionally codified).

If we are not hesitant to go back to Vesalius and Borelli, we should likewise be willing to go back to Aristotle and the T’ai Chi classics.

Machines and their parts are engineered to optimize specific tasks, and an extension of the machine conception of the organism/MCO has been the application of engineering principles in orthopedic and sports biomechanical analysis (hereafter referred to as “biomechanics”), where Borelli’s reductionist and pre-Newtonian understanding of how an organism is affected by and deals with internal and external forces is routinely assumed, and the laws of motion are, for convenience, ignored (Biotensegrity Archive Director, 2020).

Given the above problems and other known chasms between living organisms and machines, how can machine-led biomechanics provide a sound theoretical framework for understanding human movement? Discrepancies between tenets of the current day and the reductionist practice of imagining organisms as machines invite a review of long-held assumptions about the workings of living systems, including human locomotion. But, knowing that the second law of thermodynamics, the theory of evolution, systems science, and the laws of motion all pull the rug out from MCO models, what is the nature of the floor we are left standing on?

Here, we reassess persistent assumptions about locomotion based on reductionist engineering and machine models. These include Ibn Sina’s 1,000 + year old concept that muscles move the passive bones (Avicenna and Gruner, 1973), the 17th century model of the organism as a machine (Borelli, 1680), and pre-Newtonian biomechanics which fail to recognize that only external forces resulting from interactions with objects within an organism’s environment can change a body’s spatial position (Newton et al., 2006).

In search of a better way to understand locomotion (swimming, walking, running) than what machine models can give, we looked for the most energy conserving models, as 2LT is the algorithmic imperative that directs an organism’s choices (Gracovetsky, 2018; Nicholson, 2018). We investigated compatible concepts emerging within the broader field of biology and organismic movement, including ecological psychology, processual biology, soft matter and biotensegrity. We also looked for inspiration from areas both modern and ancient. On the more modern side, inspired by the ecological perspective, we explore new modeling and visualization opportunities based on behaviors, actions and relations, free of cadaveric anatomical representations and assignments (Kugler and Turvey, 1987). In the area of practical experience, we also looked at the cultural wisdom of movement traditions such as the martial arts, dance, sports and performance, since, just as with biology in general, these specialized practices are constrained to be sustainable, and to work (Lander, 2013).

Centering on the constraints necessitated by the second law of thermodynamics, the theory of evolution, a systems science view of organisms and the laws of motion, we set out to rethink organismic biomechanics to create a fresh theory of locomotion. The result is an updated, energy efficient “bouncing bones” (BB) model for human and animal locomotion which jibes with the current science. In a BB scenario, the body receives and stores energy during walking and running, tapping the ground reaction force and redirecting it through the body’s center of mass, which then governs the body’s movement. Not surprisingly, the BB model also enjoys compatibility with emerging sciences, and unites modern science with ancient wisdom, instinct and practical experience of movement.

## 2 The current scenario

Locomotion, such as walking and running, is such a fundamental process, one would think that, by now, it would be thoroughly studied and well understood. Not so (Mayer, 2020).

Although machine models have provided insights that have led to beneficial results in, for example, the invention of medical devices, the established and energetically compromised inverted pendulum model widely applied in biomechanics, along with its many permutations, is fraught with outmoded assumptions, opening an opportunity for a course correction which explores alternative models (Kuo et al., 2005; Kuo, 2007). Currently accepted models of locomotion are engineering frameworks based on machines, and this centuries-old metaphor for organisms cannot be reconciled with fundamental tenets of modern science established after the MCO became a fixture, including Newton’s laws of motion (1728), the second law of thermodynamics (1824), the theory of evolution (1859), and a systems science view of organisms (1954).

Calculations of assumed forces derived by employing inverse rigid-body dynamics and static free-body diagrams are unsuitably applied to the dynamical processes of synergistic organisms. We can completely take apart a bicycle or an F/A-18 Hornet and then put it back together again, and they will work just fine, but the Humpty Dumpty problem of old tells us from the time we are children that living organisms just don’t work that way (Dorit, 2017). Living bodies are not machines with discrete parts, but holistic, non-fractionable systems (Bertalanffy, 1969; Prigogine and Nicolis, 1985; Turvey, 2019; Kondepudi et al., 2020). Moreover, the movements and behaviors of organisms are never linear or machine-model precise (Levin, 2014; Biotensegrity Archive Director, 2020; Kelty-Stephen et al., 2023; Mangalam et al., 2023; Mangalam et al., 2024).

## 3 Flaws of presently accepted models

### 3.1 The inverted pendulum

The long running standard for walking has been the inverted pendulum model, which imagines an upside-down pendulum standing on its pivot point (Alexander, 1992). In a standard pendulum, once we impart some energy into the system, gravity (a necessary component), acceleration, and momentum take over and, with only the smallest external input to overcome a bit of air resistance and friction, the periodic motion can go on indefinitely (just think of pushing a child on a swing). But an *inverted* pendulum is an unstable, energy depleting, self-defeating device (Kuo et al., 2005). It has no periodic motion in any sense (Kelty-Stephen et al., 2023; Mangalam et al., 2023). Each “swing” is actually a fall in which it loses all of its energy, requiring a new input of external energy to raise it to its apical position; only then can gravity take over and bring it to its perigee. It must stay there, at rest, until another external force hoists it up again. This energy-intensive proposition is an extension of the comfort traditional biomechanics has with MCO models.

### 3.2 The spring mass model

The spring mass model (SMM) for running and walking is a newer standard in biomechanics (Blickhan, 1989; Sawicki et al., 2009; Koepl et al., 2010). The SMM is a modified inverted pendulum model where potential energy, rather than originating in the muscles, is thought to be gathered from the ground reaction force (GRF), and stored in the tendons and fascial tissue. This then is seen as powering the Achilles tendon to plantar flex the ankle and provide “push-off,” levering the body mass and catapulting it into the air. The GRF received each time the foot strikes the ground loads the internal springs — in this case seen as the muscles and tendons — and allows the organism to continually gather new energy.

Koepl, et al. apply the SMM to walking as well as running (2010). Although not problem-free, this is a much more energy efficient system than imagined with the standard inverted pendulum, and it is more consistent with Newton’s Laws, as the energy source needed to accelerate a body comes from an external source rather than an internal source. The body is still an inverted pendulum, but there is now a watch spring, wound by gravity, making it tick.

When the SMM was named and proposed by Blickhan in 1989, the general concept was already in the literature. Alexander, for example, suggested that most large animals use stored elastic energy and that (at least) some small animals do as well. He described how the kangaroo, and the kangaroo rat, store elastic energy in the Achilles tendon (Alexander and Vernon, 1975; Biewener et al., 1981). In Alexander’s estimation, this could be used to return 70% of the energy needed for propulsion (Biewener et al., 1981).

Since it posits that an external force accelerates the body mass rather than attributing accelerating forces to muscles, the SMM accords with Newtonian laws of motion by using an external force, the GRF, to move a body at rest, a congruence not found in the standard inverted pendulum model. But there are still a few problematic embedded contradictions (and here we will present only a sample).

First, the body mass and its parts are accelerating to the ground at 9.8 m/s^2^, even when we are standing quietly, and our feet are connected to the ground by gravity. Increasing tension in the Achilles tendon can only pull the body mass down toward the gravity-anchored foot. The internal muscles would be powerless to resist the buckling and collapsing of this near-frictionless jointed structure, as they are part of the falling body and only an external force can stop the fall. Rather than being catapulted into the air, tensioning the Achilles tendon would accelerate our already-falling bodies and bring us crashing to the ground.

Secondly, GRF is a vertical force: it can only push up, not horizontally. This is expressed in Newton’s third law, but it was also recognized by Aristotle (2006), who pointed out, “as the pusher pushes so is the pushed, and with equal force.” In the usual SMM, forward motion is assumed to be created by shear forces underfoot, but if this was the case, there would be evidence in the footprints of runners on the beach. It is easy enough to observe footprints from a number of species over time, including humanoid, in readily available fossil images. Over millennia, evidence of significant shear is almost never seen. Underfoot shear force would also be a braking force, slowing or stopping forward motion and wasting energy, putting the organism in conflict with the second law of thermodynamics.

A third point is, as envisioned in a SMM, the body’s springs are thought to be acting in *series (1/K1 + 1/K2 + 1/K3 … )*, like links in a chain (Putnam, 1993; Calabrese, 2013). We know that a chain is only as strong as its weakest link: because there are no redundancies in a series linkage system, any weak link could bring the entire system down. Preferable for resilience and endurance in a system would be for the springs to work in *parallel* with one another, and this is what we propose is happening. In a parallel system (*K1 + K2 +K3 …* ), groups of springs work together, distributing loads and building in redundancies to manage stress and strain. It also allows for a variety of alliances and reconfigurations to be established and relinquished as needed, such as in the soft-assembling synergies described by Fultot et al. (2019), Profeta et al. (2020), Biotensegrity Archive Director (2020), and Lowell de Solórzano (2020). This protects the system as a whole from courting failure due to local force concentrations.

Over the centuries, engineering principles have been applied to a parade of revisions on a model where muscles are imagined to move inert bones, people can pull themselves up by their own bootstraps, centers of mass can change inertia without outside help, and forces can be mathematically resolved within the system despite its being comprised of unmeasurable internal components with innumerable degrees of freedom (Levin et al., 2017; Lowell de Solórzano, 2020; Profeta et al., 2020). But organisms evolved quite independent of human engineering sensibilities, and cannot be expected to adhere to them. What if, instead of yet again trying to tweak an old model built on assumptions stacked up like pancakes, we were to start fresh and reason this out through first principles, what would the governing considerations be, and what kind of models would we need?

We will address the last question first.

### 3.3 Models in general

Models are representations, and scientific models come in a wide variety, including metaphorical, physical, graphical, theoretical and conceptual, and statistical, mathematical and computational; plus, there are hybrids involving two or more of these. Conventional biomechanics models, despite their longstanding utility, are flat, graphical hybridizations based on cadaver dissections and machine schematics, factors which carry with them obvious limitations for modeling living organisms.

We are not here to simply criticize existing models. We also champion efforts to discover new models to illuminate new perspectives. Scientific models are not valued for their accuracy in representation, so much as for their usefulness in terms of insights and understanding. We need new models thet can elucidate aspects of living, dynamic, ever reconfiguring organisms.

In this, we are mindful of the words of George E. P. Box: “Essentially, all models are wrong, but some are useful,” since the perfect model does not exist, because it would have to be an exact duplicate of that which we seek to model (Box and Draper, 1987; Mangalam, 2024). We also want to highlight several points made by Kugler and Turvey (1987), who wrote that “The structures that support an action should not be confused with the action,” and that “movements and their units are not things, but relations.” An organism’s movements and actions are “not a particular aggregation of elemental anatomical mechanisms, but a *specific mode of resource use,*” whose “variability is in reference to preserving the function it fulfills rather than preserving any particular aggregation of body parts that it happens to involve” (Kugler and Turvey, 1987).

Since, according to Kugler and Turvey, actions and their related movements are, “functionally specific, not anatomically specific,” (1987), the models we seek are not necessarily anatomical, but are selected for the purpose of stimulating insight into this or that aspect of *behavior* (springs, tensegrities, a flying tennis racket), *function* (chains, eggs, Anglepoise lamps), *relationships* (Baron Munchausen, pushing a child on a swing, a ship pulling its tender) and *resource use* (a Slinky helical spring toy, a watchspring).

## 4 First principles

In reasoning from first principles, we start with the second law of thermodynamics, interlinking evolution, systems science and the laws of motion. We then look for congruencies in ideas about locomotion from the wider field, including traditional movement wisdom. For example, both physics and the T’ai Chi classics tell us that movement comes from the center (Itcca, 2023; Lewin, 2015), and Aristotle seems to have agreed, having written, “the original seat of the moving soul must be in that which lies in the middle, for of both extremes the middle is the limiting point” (Aristotle, 2006).

We are not the first, nor the only ones, to begin an exploration of human (or even organismal) locomotion from first principles, and we realize that our chosen departure point linking 2LT, evolution, system science and the laws of motion is just one possible choice for rethinking human movement. Thus, we encourage the reader to investigate more deeply systems science, processual biology and ecological psychology for very different, yet we believe completely compatible, approaches to the common challenge presented in encouraging scientific investigation to move away from the MCO. And this is just the point, because the more independent cohorts take a stab at this beast, from their many different yet completely valid directions (and, others out there, please step forward!), the more the old paradigm will finally be understood to be unsustainable. Just as with Kuhn’s analysis of the gradual shift from belief in an earth-centered planetary universe to the heliocentric perspective we have today, the lack of sustainability of an entrenched model will need to be evidenced repeatedly in order for a new paradigm to be allowed to emerge (Kuhn, 1962).

We do find it a potential obstacle to the development of productive scholarly conversation if *accepted* models are regarded as contemporary, even if anchored in unexamined concepts going back hundreds (and sometimes even thousands) of years, while equally ancient wisdom which finds compatibility with more modern conceptual areas (systems science, processual biology, ecological psychology, soft matter, biotensegrity) and therefore may be of considerable worth, if *unaligned* with mainstream entrenched concepts, tends to be undervalued and overlooked. Especially when we have had this wisdom in our academic canon for, in some cases, centuries, we should be open to taking another look.

### 4.1 Evolution and the second law of thermodynamics

As we have stated from the beginning, there is no getting around the second law of thermodynamics. As we have also seen, when it comes to organisms, 2LT is inseparable from the constraints recognized in evolution and systems science. Join the principles of these interlinked areas of study with the equally unavoidable laws of motion, and we have a sound set of first principles to reason from.

### 4.2 Systems science

In complex systems such as organisms, it is only the whole that counts.

The synergistic interactions of component parts in complex systems dictate that the whole is greater than the sum of its parts, and studying sub-systems out of context with their systems may be deceiving (Fuller and Applewhite, 1975; Hatze, 2002; Huijing and Baan, 2008; Prigogine and Stengers, 2018; Profeta and Turvey, 2018; Profeta et al., 2020; Westlin et al., 2023). Although we can use biological solutions to inspire engineering in biologically informed disciplines, thus far, we can never completely reverse engineer the indissoluble wholeness of life.

In a system, it is only by understanding how a part functions within the whole that it can be analyzed, and then only in a limited way as the complexity cannot be entirely unraveled. The application of reductionist principals and models imagining the body to be a machine are out of step with the current understanding of organisms as complex dynamic systems operating far from equilibrium (Bertalanffy, 1969; Prigogine and Nicolis, 1985; Hatze, 2002; Nicholson, 2018; Mangalam et al., 2023; Mangalam, 2024). The MCO imagines organisms to be linear, fractionable assemblies; but we know that organisms cannot be taken apart and then reassembled without dire consequences. Remember Humpty Dumpty.

### 4.3 The laws of motion

**…**and neither would there be any walking unless the ground were to remain still, nor any flying or swimming were not the air and the sea to resist. — Aristotle; On the Movement of Animals

The laws of motion tell us that an object, or a body, cannot change its inertia on its own. That means that if a body is moving along, there is nothing it can do by itself to speed up, slow down, or stop. It must have outside help, an external force.

Aristotle observed this and put it this way, “this which resists must needs be different from what is moved, the whole of it from the whole of that, and what is thus immovable must be no part of what is moved; otherwise there will be no movement” (Aristotle, 2006).

Similarly, we cannot walk or run in space. It is the force of gravity created by an external mass (a mass big enough to push against), that gives us the power to change our spatial position on Earth. Without it, we are helplessly immobilized. For astronauts, it is the interaction with the mass of the spaceship that provides the external counterforce necessary to push buttons and move levers on the spaceship. In microgravity, we can change the shape of our body but we cannot create any significant positional change for ourselves without pushing against an object that is of a comparable or greater mass than our body’s.

Although Newton’s laws are established and accepted across the sciences, most biomechanical models of locomotion assume it is possible for *internally* generated forces to move us forward. Examples include instances (seen in both the inverted pendulum and variations on the SMM) where the foot is imagined to act as a lever to catapult the body up into the air and forward in space (Blickhan, 1989; Sawicki et al., 2009; Koepl et al., 2010; Pàmies-Vilà and Font-Llagunes, 2014; Sawicki and Khan, 2016). This is akin to Baron Munchausen pulling himself up out of the mire by his own hair, or lifting ourselves off the ground by pulling on our own bootstraps (Münchhausen Trilemma, 2023).

## 5 Updating and upgrading the model

Bianchi et al. (1998) suggest that an organism’s use of a gravity-based, body-centered reference frame for the control of intersegmental coordination would represent a natural choice for implementing the exchange between gravitational potential energy and the forward kinetic energy of the body. Matthis and Fajen point out that during locomotion, “the locomotor system harnesses and redirects these passive mechanical forces in order to generate a stable and efficient walking gait” (2013). Extending from these, we can envision a systems model for locomotion that is both energy efficient and consistent with Newtonian laws of motion and gravity.

### 5.1 The body’s springs

The spring mass model is a good start for modeling locomotion, as the springiness of the body has long been recognized, and from an energetic standpoint, the body could increase efficiencies by making use of that springiness.

L. Carnot saw the body as interconnected springs from cell to organism as early as 1803, and the literature is replete with discussions of springiness at multiple scale levels in various organisms (Carnot, 1803; Roberts and Azizi, 2011; Knight, 2021). An interconnected scaffolding of springs would include the bones, which (as any orthopedic surgeon will attest), are very springy. Somatic educator Mabel Elsworth Todd also noted the steel-like elasticity of bone (1977) and, as the stiffest springs at the macro scale level of the body, bones would be the most amiable internal structures for storing the potential energy needed for ambulation (Fish, 1993).

Muscle testing *in vitro* always assumes muscles must significantly shorten or lengthen when actively functioning, but *in vivo,* it is known that muscles often act isometrically, perhaps more like the springs in an Anglepoise lamp, and muscles are weakened by any significant change in muscle length (Cleland, 1867; Lombard, 1903; Levin et al., 2017).

Alexander and Dudek both considered legs as springs, but emphasize only muscles and tendons (Alexander, 2023; Dudek and Full, 2006). Knowing that the stiffer the spring, the springier it is, we should not ignore the bones.

Alexander did think about it: “Because leg bones bend only by amounts that seem so trivial, I am inclined to think their proportions more likely to depend on being strong enough, than on the need to be stiff enough” (2003).

Perhaps they were not aware of the possibility of a systemic and structural tenso-compressional relationship, wherein tensional members are under tension and compressional members are under compression, such as in a tensegrity structure. Tensegrity structures were only devised in the last century, and are still not fully understood, but, particularly when their tension cables are inextensible, they have surprisingly powerful elastic behaviors which are “greatly multiplied by the geometry of the system,” (Ucpress, 1976).

In stiff compression springs, small amounts of compression store much more energy than the same amount of movement creates in more compliant springs, so the compression of bones working in parallel may be hardly measurable, yet still powerful. The same principle applied to tension springs allows even minimal shortening and lengthening of muscles to be highly effective (Wikipedia, 2023).

We know that all bones are under compression, as is set out in Wolff’s law, but also because when long bones break (for example), the pieces tend to overlap, demonstrating that previous to the breakage they were managing compressive forces, which is why limbs often must be tractioned in order for bones to be set and to heal. This, plus their shape and position suggest that especially the long bones could function like leaf springs. Correspondingly, we know that softer tissues are under tension, because when they are cut, they pull apart. If, as we suspect, these components function in tandem, as in a tensegrity system, and therefore in parallel, their behavior could be bouncy and springy well beyond what might otherwise be anticipated by the individual materials themselves.

### 5.2 The center of mass

*Accordingly it is plain that each animal as a whole must have within itself a point at rest, whence will be the origin of that which is moved, and supporting itself upon which it will be moved both as a complete whole and in its members*—*Aristotle; On the Movement of Animals*.

How can a body at rest (we will use a person in our model) initiate and maintain forward motion when the GRF can only produce a vertical force? It happens as we get ready to take our first step, and its locus is in our center of mass.

The T’ai Chi classics teach that the body’s movement is led by the center, approximately what a physicist might call the center of mass (CoM) (Itcca, 2023). Likewise, physics tells us that the movement through space of a body in motion is governed by its center of mass, and that bodies behave as if all their material is concentrated as a point mass in the CoM (mittechtv, 2009; Lewin, 2015). Once movement is imparted to a body’s CoM, all other parts of the body follow along like a ship pulling its tender, whether the structure is inanimate or animate. This has been demonstrated robotically, such as is discussed as passive dynamic walking in bipedal robots, but has also been shown in other robot models such as IHMC’s HexRunner (Collins et al., 2005; Matthis and Fajen, 2013; Michelle Thorpe, 2019). This means that once a body’s springs are loaded, the center of mass takes over and movement is centered around the CoM and follows its lead (Gracovetsky, 1997; mittechtv, 2009).

All masses have a gravitational pull, so opposing masses are in competition for control of any mass caught between them, evidenced by the ocean tides caught between the pulling forces of the Earth and the Moon. Just as in a “Zero-gravity” flight experience, a falling body is effectively weightless (though not massless), because the force of acceleration counterbalances the force of gravity. For a given body in this situation, each particle in the mass is no longer being governed by Earth’s gravity but only by the gravity of the mass of the object of which it is a part. Just as each grain of sand is moving as part of the Earth’s CoM, each part of a moving object rotates around the object’s CoM. Standing on Earth, we are moving at roughly 1,000 mph, but we don’t notice it at all.

To gain forward motion from a standing position, by leaning forward even slightly, we move our CoM out of line with its support base, and the body begins to fall forward. This fall forward puts the CoM on a diagonal path towards the ground ahead of us, a path that can be understood as the resultant vector of the downward pull of gravity and the forward motion generated by the leaning. Newton’s first law of motion tells us that once we are moving forward, no additional impetus is needed in order to keep going in that direction.

### 5.3 Putting it all together

Walking as controlled falling is a recognized concept and although generally it has not been integrated into movement models based on the SMM, this is easily done (Lacquaniti et al., 1999; O’Connor and Kuo, 2009).

The force transmission we envision is through interconnected networks of *ad hoc* assemblages and soft-assembled synergies of closed kinematic chain linkages at multiple scale levels, including the stiffening muscles, which function in parallel and allow the body to become loaded by external forces as a *whole system*. Then, like a popping up Jack-in-the-box, a body can release its stored internal power for explosive action to generate propulsion (Lee et al., 2014). After initiating a forward lean, falling on one’s face is blocked by the (previously rear) foot being pulled directly ahead.

Like a Slinky going down the stairs, whose last few coils are pulled forward and in towards the CoM with such force that (following Newton’s First law) they fly right past it and down onto the next step, the back leg, unless actively constrained, is pulled forward at minimal energetic cost (Kugler and Turvey, 1987; Simonsen, 2014; Brown University Department of Physics, 2022). As the foot contacts the ground, our forward moving CoM lines up with it, and the kinetic energy of the GRF (the body mass, *m*, multiplied by the velocity squared, mv2), enters the body and loads our springs, imparting potential energy to them.

The bones are primarily compression springs, and the tendons, ligaments and other softer tissues are primarily tension springs, and their tensegral configuration (as described above) allows both kinds of springs to be loaded mutually, just as bow strings interact with a bow. Release of the stored energy creates an acceleration of the CoM equal and opposite to the GRF, straight out from the center of the earth. The body is propelled upward by the GRF, and forward by the momentum of the CoM created by the forward tilt of the body.

As the body’s joints are near frictionless, then, except for wind resistance, the forward motion of the CoM is not impeded as the body glides effortlessly over its near-frictionless joints, with only the foot on the ground pausing briefly to interact with and receive the vertical forces from the ground while the parts above the planted foot continue on their way propelled by the inertia of the CoM (Gouttebarge et al., 2015).

The body’s springs, particularly the stiffer springs that are energized by compression, store the potential energy and then release it as a force that propels the mass. That force is focused in the CoM so that the body moves as if all its mass were in that point and the body’s component parts follow (Dudek and Full, 2006; mittechtv, 2009; Lewin, 2015). Having done the job of transferring energy to the CoM, the limbs are then lifted from the ground as the CoM moves upward away from the ground. Forward motion initiated by a slight forward tilt of the body is maintained.

By borrowing energy from the body’s interaction with gravity, organisms can minimize their energy expenditure and maximize their efficiency, in keeping with the second law of thermodynamics. As we previously noted, Nature, working through its handmaiden, evolution, will do just that.

### 5.4 Historical perspective

Over the centuries, humans did what they could to learn from the pieces they found or dissevered, and imaginations tried to make mechanical sense of anatomical discoveries. Western scientific understanding of anatomical function may be traced back at least to Galen, who, living in a culture where human cadaver dissection was forbidden, availed himself of dead gladiators and bodies that washed ashore after a flood (Newberry, 2023). Vesalius was allowed to dissect, and achieved a new level of anatomical documentation, but his guesses about the functional processes of the previously living pieces he cut out were necessarily lacking; for example, his idea that muscles have “beginning” and “end,” or “origin” and “insertion” points implies a lack of consideration for the stages of human development (Heseler, 1540).

Newton and Borelli’s time overlapped, but Newton’s laws of motion were not considered in Borelli’s De Motu Animalium. Bubonic plague reached Cambridge in the summer of 1665, and Trinity College closed for 2 years. Newton, like so many others, returned home. There he had his *annus mirabilis,* during which he theorized about calculus, his laws of motion and more, much of which he published later in his Principia (presented in 1686 and published in 1687) —too late for Borelli (interestingly, Newton knew of, and referenced, Borelli’s work, such as on the movement of planets).

Borelli’s book depicts machinery within us that we know is not actually there, often depending on external counterweights we obviously don’t have. It asks us to believe that forces can be resolved into these imagined internal component parts (Alexander, 2003; Hatze, 2002). His anatomical assignments proved so attractive that they have persisted in the literature without much review, despite being at odds with advances in scientific understanding that developed across the intervening centuries (which may have started with Newton’s laws of motion, but which eventually included the second law of thermodynamics, the theory of evolution, systems science, and more).

Regardless of its persistence, the reductionist MCO has not gone unchallenged. Alexander Pope, a contemporary of Newton, wrote, “Like following life thro’ creatures you dissect, You lose it in the moment you detect” (Pope, 2022). The French physician, Chaptal, in a speech to medical students in 1796, declared, “He will never be a doctor who isolates the human body to better study its functions: when he thinks he knows the man, he will only know his corpse (Chaptal, 1796). About one hundred years later, Cleland, and then Lombard, presented research countering the Borelli model (Cleland, 1867; Lombard, 1903). Their admonitions were all but lost in the vicissitudes of time. In this century, Dupré, Hatze, Kugler, Levin, Mangalam, Nicholson, Turvey and others have picked up the baton and the machine model is again being questioned (Dupré, 2022; Hatze, 2002; Kugler and Turvey, 1987; Levin, 2002; Mangalam, 2024; Nicholson, 2018; Turvey, 2019). This time, global communication among researchers is nearly instantaneous, there are new perspectives to consider (ecological psychology, processual biology, systems science, complexity, soft matter), and there is a compelling systems model for force interactions available (tensegrity) that did not exist a century ago.

## 6 Discussion

We cannot know all the intricacies of our inner workings, because we cannot get inside ourselves without altering the very systems we are curious to investigate. Left to our imaginations, then, we humans have tried to glean what we can about locomotion from encounters with lifeless skeletons and cadavers, embellished by insights from our mechanical inventions. We see a pile of bones. How can we get them to move? Perhaps the pieces we’ve cut out and named “muscles” do that? This story dates back at least to Ibn Sina, and, although we have no way of proving its veracity, we find it so appealing that we’ve kept it around (Avicenna and Gruner, 1973).

Of course, the question itself has it backwards. Locomotion is not a problem of how to get dead parts to move. Rather, movement is something we inherit at conception, and locomotion is an extension of that continuous movement, resulting from interaction with our environment.

There are many parts to this puzzle that have yet to be introduced, such as the fact that all living systems are part of evolutionary and developmental continuums, or the implications related to the transmutable soft matter nature now recognized in all biological organisms (Gracovetsky, 1997; Poon and Andelman, 2006; Marx, 2020; Miyazaki et al., 2022). To this last point, for example, it is known that the stiffness of bone changes with the rate of loading, and that biologic tissues have nonlinear stress/strain curves (Hamley and Castelletto, 2007; Novitskaya et al., 2011).

The BB model makes no assumptions about internal postural control, is not a predictive model for sensorimotor control, and should not be seen as assuming or dependent upon an engineered, machine type of periodic movement which is linear and infinitely replicable. We are not talking about gait cycles, nor attempting to model the specifics of stride length or rate, nor of stride regularity or regulation. Our focus is on the physical possibilities for interactions between the organism and the earth, and the physical possibilities for an organism’s recruitment, distribution, storage and management of external forces.

We do not speculate about how any specific movements are controlled internally by the body’s many interlinked systems. In this, although we get there through very different pathways, we hold with Mangalam et al. (2024) that organisms are “black boxes” of internal force management, with an incalculable number of internal degrees of freedom at their multiple scale levels of interlinked, ever-reconfiguring heterarchical coalescences (Levin et al., 2017; Biotensegrity Archive Director, 2020; Lowell de Solórzano, 2020). Organisms are necessarily nonlinear, non-ergodic systems whose stability is anything but static, but rather is a *result* of their mobility, as well as their ability to (within certain limits) constantly adapt and reconfigure (Lowell de Solórzano, 2020; Mangalam et al., 2023).

No two steps are exactly alike, any more than any two breaths, or any two individuals are. And here we link to ancient wisdom once again, as this correlates directly with Heraclitus’ insight that, “Everything flows,” and that “No man ever steps in the same river twice” (Heraclitus, 2024). The simple model proposed here only offers a sketch of the complex functions that we call walking and running, but our Bouncing Bones modification of the SMM brings us a step closer to actuality.

This article is bereft of mathematical calculations, recognizing that calculations of internal force resolutions are not the same as actual measurements and acknowledging that models for animal locomotion based on inanimate machines will have inherent inaccuracies. Internal pressures can be measured (blood pressure, arterial pressure, lung pressure, spinal fluid pressure, bladder pressure, intervertebral disc pressure, etc.), but pressure only measures the force on the walls of the pressurized container, it does not indicate the force which that container exerts on structures external to it (for example, the pressure inside an automobile tire is independent of the weight of the vehicle itself, and not related to the force the weight of the vehicle exerts through the tire onto the road beneath it).

Internal forces in tissues cannot be measured directly, so we cannot know their direction (Hamant and Traas, 2010). In the present state of the art, muscle forces and compression loads across joints can only be calculated, not measured, and that is most typically done using inverse dynamics and free body diagrams which are incompatible with living biosystems (Panjabi and White, 2001; Hatze, 2002; Vogel, 2013). Thus, we present here a model that is lacking calculations and numbers. This may leave those that crave tidy, numerical answers somewhat unsatisfied, but until we find a way to accurately measure internal forces and not just pressures, that discomfort may be something we have to live with.

Because the muscles act as turnbuckles within closed kinematic chain systems, and work in parallel (rather than in series), the stiffening of the joints necessary for stability is a function of both the geometry, and the processes, of closed chain kinematics (Lombard, 1903; Levin, 1981; Connelly, 2013; Levin et al., 2017; Liu et al., 2019).

Rather than the reductionist machine model standard, our BB model integrates with the increasingly (if not yet widely) accepted biotensegrity model for organisms (Caspar, 1980; Levin, 1981; Ingber, 1998; Tadeo et al., 2014; Turvey and Fonseca, 2014; Reilly and Ingber, 2018; Profeta et al., 2020; Boghdady et al., 2021; Oh et al., 2022; Berry et al., 2023; Hukumori and Nishimura, 2023; Kelty-Stephen et al., 2023; Mangalam, 2024). The biotensegrity model, generally, considers tensegrity in biology, and is conceived as an energetically and materially efficient process and force management model manifesting across an organism’s multiple scale levels. It is consistent with 2LT, evolution, and the dynamic, ecological and processual systems thinking of Bertalanffy, Nicholson, Turvey and others (Bertalanffy, 1969; Hatze, 2002; Turvey and Fonseca, 2014; Gracovetsky, 2018; Nicholson, 2018; Dupré, 2022).

Under ideal conditions (no headwind, perfectly resilient surface, level ground, barefoot), the BB model accommodates reconfiguring of the body as it is falling from its apogee using muscles as part of its closed kinematic chain system. Here, the muscles take on the role of stiffening the system as the springs are loaded, and muscles working isometrically and isotonically would hardly be working at all. In keeping with the second law of thermodynamics, walking and running would require minimal energy expenditure.

Interestingly, this aligns with traditional movement philosophies. While western anatomists and physiologists focused on how metabolic activity can create enough internal power to fuel the motor of an energy intensive machine body sufficient to drive external movement, students of eastern movement systems, such as Tai Chi, Yoga, Aikido, Thang Ta, Wing Chun and similar arts, cultivated the use of “least effort,” harnessing external forces to create body movement—a more holistic and energy efficient model.

Of course, ideals are never fully realized, but that doesn’t mean the organism isn’t always doing its best to conform to the second law of thermodynamics. How could this not be the case?

## Data Availability

The original contributions presented in the study are included in the article/supplementary material, further inquiries can be directed to the corresponding author.
